# Krüppel-Like Factor 10 participates in cervical cancer immunoediting through transcriptional regulation of Pregnancy-Specific Beta-1 Glycoproteins

**DOI:** 10.1038/s41598-018-27711-8

**Published:** 2018-06-21

**Authors:** Daniel Marrero-Rodríguez, Keiko Taniguchi-Ponciano, Malayannan Subramaniam, John R. Hawse, Kevin S. Pitel, Hugo Arreola-De la Cruz, Victor Huerta-Padilla, Gustavo Ponce-Navarrete, Ma. del Pilar Figueroa-Corona, Laura Gomez-Virgilio, Teresa I. Martinez-Cuevas, Monica Mendoza-Rodriguez, Miriam Rodriguez-Esquivel, Pablo Romero-Morelos, Jorge Ramirez-Salcedo, Michael Baudis, Marco Meraz-Rios, Florinda Jimenez-Vega, Mauricio Salcedo

**Affiliations:** 1 Laboratorio de Oncología Genómica, Unidad de Investigación Médica en Enfermedades Oncológicas, Hospital de Oncología, CMN-SXXI. IMSS, CDMX, Mexico; 2 Departamento de Biomedicina Molecular, CINVESTAV, CDMX, Mexico; 3 Department of Biochemistry and Molecular Biology, Mayo Clinic, Rochester, MN USA; 4 Unidad de Microarreglos de DNA, Instituto de Fisiologia Celular, UNAM, CDMX, Mexico; 5 Institute of Molecular Life Sciences, University of Zurich, 8057 Zurich, Switzerland; 6 Laboratorio de Biotecnología, Universidad Autonóma de Ciudad Juárez, Ciudad Juárez, Chihuahua Mexico

## Abstract

Cervical cancer (CC) is associated with alterations in immune system balance, which is primarily due to a shift from Th1 to Th2 and the unbalance of Th17/Treg cells. Using *in silico* DNA copy number analysis, we have demonstrated that ~20% of CC samples exhibit gain of 8q22.3 and 19q13.31; the regions of the genome that encodes the KLF10 and PSG genes, respectively. Gene expression studies demonstrated that there were no alterations in KLF10 mRNA expression, whilst the PSG2 and −5 genes were up-regulated by 1.76 and 3.97-fold respectively in CC compared to normal tissue controls. siRNA and ChIP experiments in SiHa cells have demonstrated that KLF10 participates in immune response through regulation of IL6, IL25 and PSG2 and PSG5 genes. Using cervical tissues from KLF10^−/−^ mice, we have identified down-regulation of PSG17, −21 and −23 and IL11. These results suggest that KLF10 may regulate immune system response genes in cervical cancer among other functions. KLF10 and PSG copy number variations and alterations in mRNA expression levels could represent novel molecular markers in CC.

## Introduction

Cervical cancer (CC) is currently the second most common cancer among the female population^1^. As is the case with many other cancer types, host immune system evasion, involving the failure of the immune system to resist or eradicate formation and progression of incipient neoplastic cells, late-stage tumors and micro-metastasis is problematic in CC^2^. The host immune response evasion by Human Papilloma Virus (HPV) associated CC has been reviewed elsewhere^3^. Cytokines such as Interleukin (IL) 10, IL13 and transforming growth factor β (TGFβ) are known to be involved in evasion of CC from the immune system response^3,4^. Tumor-associated inflammation and the inflammatory components present in the tumor microenvironment are also recognized as having a role in tumor development^5^. This is mediated in part through the supply of bioactive molecules to the tumor microenvironment. These include growth factors, survival factors, pro-angiogenic factors, extracellular matrix-modifying enzymes, as well as the inductive signals that lead to activation of epithelial-mesenchymal transition (EMT)^2^. This phenomenon is orchestrated by a plethora of cytokines and chemokines. In CC, cytokines such as IL6, −8^6^ and −17^7^ as well as chemokines such as CCL2^8^ are known to exert these pro-inflammatory effects. Expression of these molecules in the tumor microenvironment is tightly regulated by transcription factors.

Krüppel like factors (KLF’s) are a family of transcription factors that participate in various aspects of cellular growth, proliferation and differentiation^9^. This family is characterized by three C_2_H_2_-type zinc finger domains that bind to either CACCC elements or GC boxes in the promoter of target genes to regulate transcriptional activity and gene expression^9^. Recently, we have reported the expression of several members of the KLF family in CC including KLF10^10^. KLF10 can function as a *trans*-activator or –repressor^11^. It has been previously reported that KLF10 is expressed in several tissues and various cell types that include smooth muscle, heart, glial cells, fibroblasts, myeloid cells^12^, and lymphoid cells^11^. Gene-targeting studies have implicated important roles for KLFs in immune and hematopoietic cell biology. For example, in CD4^+^CD25^−^ cells, KLF10 is known to play an important role in T-cell activation and differentiation as well as Treg suppressor functions through regulation of TGFβ^11^. It has been reported that KLF10 can also directly regulate the pro-inflammatory cytokine IL12p40^13^.

In addition to the above-mentioned functions of KLF10 in multiple cell and tissue types, no one has implicated a role for KLF10 in CC. In this report we have analyzed the molecular alterations of KLF10 in CC and have identified potential KLF10 target genes by means of DNA copy number variation (CNV) and mRNA transcription using microarrays, *in silico* analyses and putative transcriptional regulatory networks using gene knock-down approaches *in vitro* and *in vivo*.

## Results

### Genome copy number alteration in cervical cancer, 8q22.3 and 19q13.31 gain

To detect new genes associated with immune response in cervical cancer, our first approach was to identify cytogenetic regions harboring DNA gains. The results of our studies revealed that there were representative regions such as 1q, particularly 1q32.1 where the IL10 and IL24 genes are encoded, with over representation in ~23% of the CC samples. As expected, chromosome 3 showed mostly gain of DNA material. The most altered regions in the chromosome were at 3q26 and 3q21 where ~60% of the CC samples showed gains in DNA material. Recently, we reported that a tumor suppressor gene in CC, CRBP1 was located in the same region^14^.

Interestingly, chromosome 8q22.3 where KLF10 gene is localized was also gained in ~20% (22/115), lost in ~7% (8/115) and unchanged in the remaining 73% (85/115) of the CC samples.

**Figure 1:**
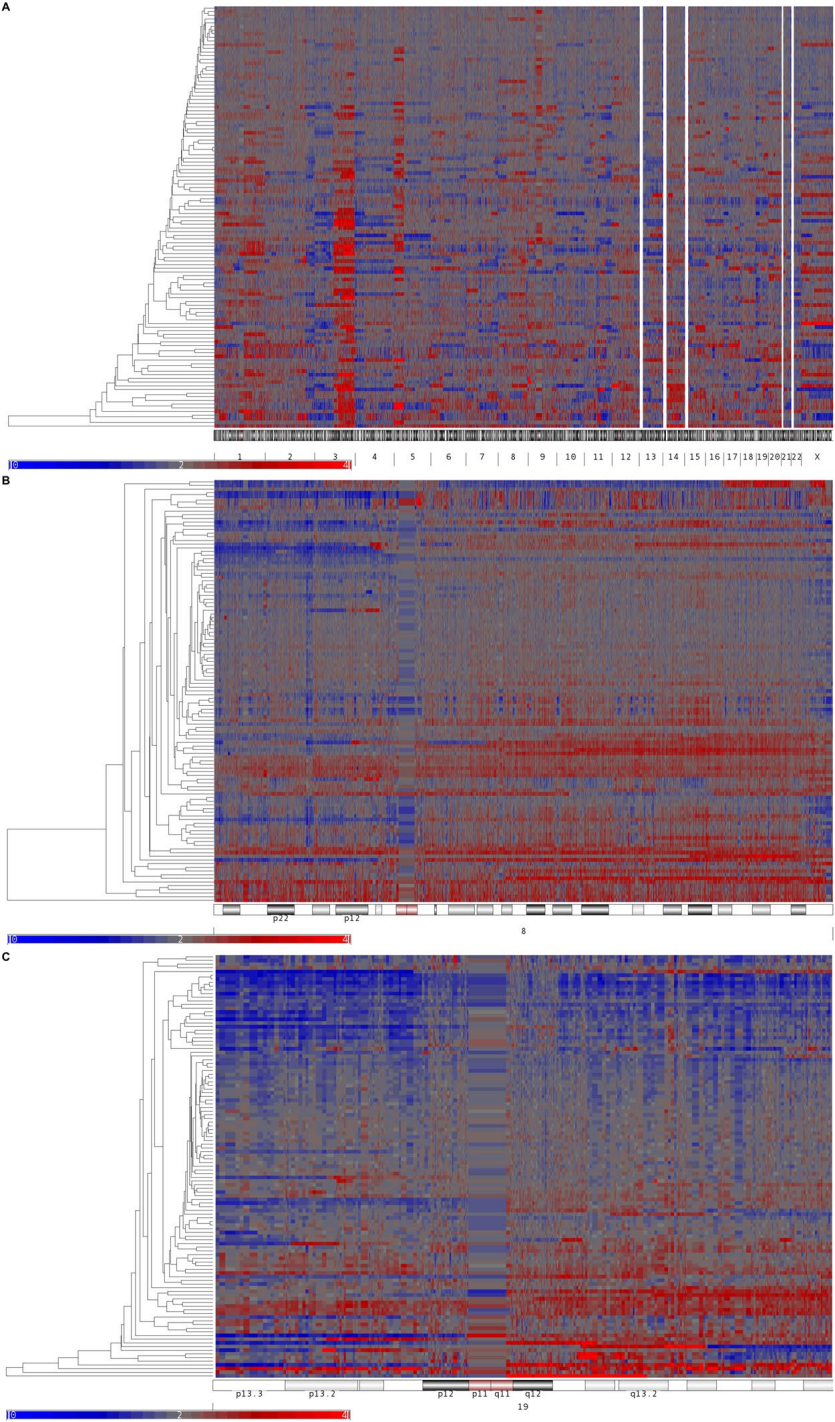
Chromosomal imbalances in CC. Panel (A) hierarchical clustering indicating genomic DNA gains in red and losses in blue in the 22 somatic chromosomes and the X chromosome in 115 CC samples. Cervical cancer genomes show gains in 1p, 3q, 5p, 20, and 8q22.3 and 19q13.31 cytogenetic regions. Panel (B) and Panel (C) indicate the hierarchical clustering of the 8 and 19 chromosomes across the CC samples analyzed. In panel (A) white stripes in the acrocentric chromosomes 13, 14, 15, 21 and 22 corresponds to nucleolar organizer regions and stripes observed in chromosome 8 and 19 correspond to centromere on panel B and C.

Since immune related genes play an important role in CC, it was of interest to look for their alterations in CC. These studies revealed that the 19q13.31 region was gained. The Pregnancy-Specific Beta-1 Glycoproteins (PSG), PSG2 gene was gained in ~20% (22/115), lost in ~7% (8/115) and unchanged in 73% (85/115). The PSG5 gene was gained in ~20% (23/115), lost in ~6% (5/115) and unchanged in 76% (87/115) of the CC samples (Fig. 1). Validations of these CNV results are required.

### KLF and immune response genes exhibit altered expression in CC

Once the candidate genes were spotted by DNA gain in copy number, our second approach was to examine their mRNA expression levels using microarray analysis. For this purpose, we compared the transcriptome of normal cervix and cervical cancer tissues, by *in silico* analysis of publically available microarray libraries.

Kruppel like factors 5 and 6 were among other genes that exhibited alterations in these analyses. These two genes were previously described to be up-regulated in CC by our group^10^.

**Figure 2:**
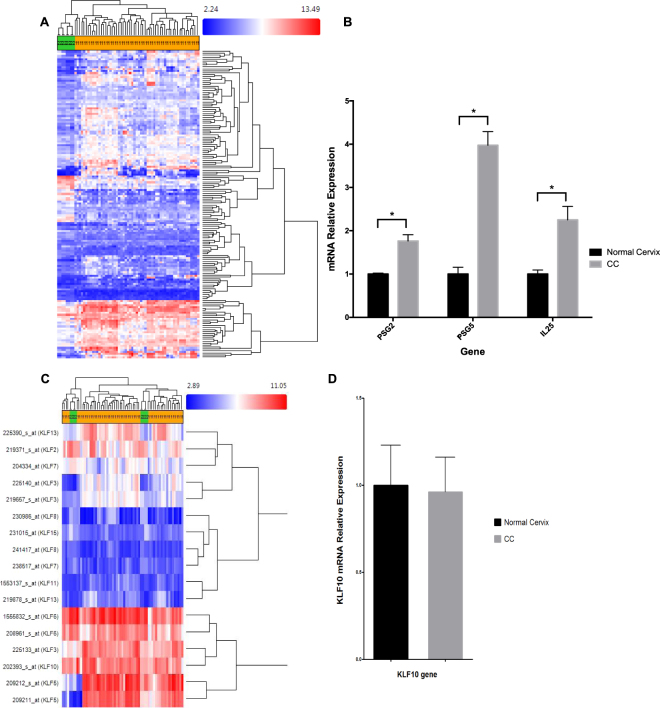
Transcriptome analysis of cervical tissue. (**A**) Heat map indicating differentially expressed immune related genes between normal and CC samples as detected by *in silico* analysis of the array datasets. (**B**) RT-qPCR validation of PSG2, PSG5 and IL25 in cervical cancer samples collected by our laboratory. Black bars represent basal expression in normal cervical tissue while grey bars represent expression in cervical cancer. (**C**) Hierarchical clustering indicating the expression levels of KLF family members in normal tissue and CC. (**D**) RT-qPCR analysis demonstrating no significant differences in the expression levels of KLF10 between normal (black bar) and CC (grey bar) tissue.

In spite of DNA amplification, KLF10 mRNA expression levels did not differ between normal and CC tissues (Fig. 2), and there where no significant clinico-pathological correlations with KLF10 (Table 1).

**Table 1:** Clinical-pathological correlations with KLF10, PSG2, PSG5 and Il25 expression.

Clinical Variables	Average ± SD	KLF10 (*p*)	PSG2 (*p*)	PSG5 (*p*)	II25 (*p*)
**Age**	51.4 ± 15.18	0.53	0.03*	0.196	0.708
≥50	12				
<50	8				
**Pregnancies Number**	4.05 ± 2.76	0.12	0.781	0.027*	0.403
≥3	13				
<3	7				
**Pregnancy natural deliveries**	3.65 ± 2.94	0.99	0.525	0.60	0.651
≥3	11				
<3	9				
**Sexual activity age onset**	18.88 ± 5.48	0.41	0.20	0.315	1.0
≥18	13				
<18	7				
**First menstruation**	13.6 ± 1.75	0.66	0.609	0.159	0.286
≥12	17				
<12	3				
**Sexual partners number**	1.7 ± 1.21	0.19	0.309	0.384	0.166
≥2	8				
<2	12				
**Tobacco consumption**		0.34	0.740	0.085	0.211
Positive	12				
Negative	8				
**Alcohol consumption**		0.34	0.238	0.018*	0.101
Positive	15				
Negative	5				
**Contraceptives**		0.79	0.38	0.056	0.525
Positive	14				
Negative	6				
**Diagnostic**		0.77	0.026*	0.011*	0.008*
CC	20				
Non-CC	3				

*Statistical significance.

Interestingly, genes from the PSG family exhibited increased expression in CC (Fig. 2). Multiple interleukins and chemokines including IL17, CXCL8, CXCL1, CXCL10 and CCL20 were up-regulated in CC samples compared to normal tissue (Fig. 2). We also observed homogeneous expression patterns for KLF3, −7, −8, −11 and −15. However, only KLF10 and KLF15 consistently exhibited copy number gains across these samples. Of these two genes, only KLF10 is known to play an important role in immune regulation.

### Up-regulation of PSG2, PSG5 and IL25 genes in cervical cancer tissue biopsies

The transcripts exhibiting altered expression in the *in silico* CC microarray analyses were further evaluated in the cervical cancer biopsies collected (20 CC and 3 normal cervix samples). We decided to validate the PSG2 and PSG5 genes, because they were found to be consistently gained in copy number and alternatively expressed in CC. Simultaneously, we also evaluated IL25 (IL17E), because several IL17 family members were up-regulated in CC samples and also participate in immune regulation. RT-qPCR analyses revealed increased expression of PSG2 and PSG5 by 1.76 and 3.97 fold respectively in CC samples, compared to normal tissues. At the same time, a 2.25 fold up-regulation of IL25 was observed in CC samples (Fig. 2). These genes could represent attractive targets for immune based therapy.

### KLF10 knock down (KD) in SiHa cervical carcinoma cells down-regulates the PSG2 and PSG5 genes

**Figure 3:**
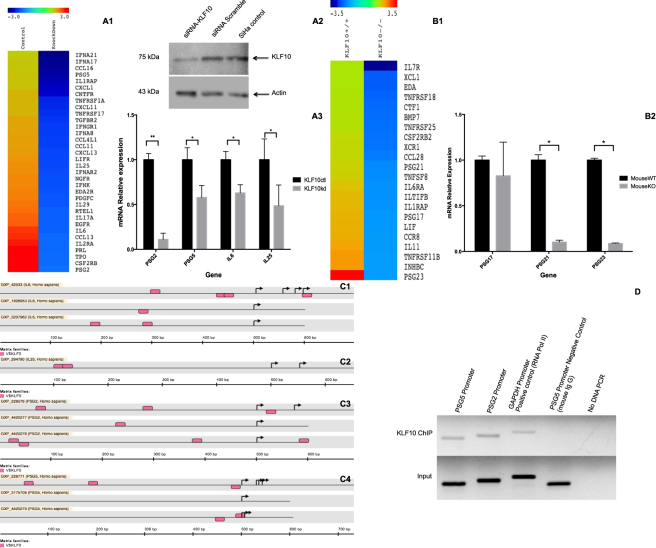
Relative expression analyses of IL25, PSG2 and PSG5 genes. Panel (A1) Heatmap depicting expression levels of indicated immune system response genes that are down regulated in SiHa cells following siRNA-mediated suppression of KLF10. Panel (A2) Western blot depicting KLF10 protein levels in the knocked down cells compared to scrambled siRNA and untreated control cells. Panel (A3) show RT-qPCR validation of the down-regulated genes in microarray data: IL6 with 38%, IL25 with 52% PSG5 with 43% and PSG2 with 89% down regulation in KLF10 siRNA treated cells in comparison with the control cells without any treatment. Panel (B1) presents heatmap corresponding to immune system response related genes dowregulated in KLF10 KO mouse compared to KLF10 WT mouse. Panel (B2) show RT-qPCR validation of PSG17 down regulation of 18%, PSG21 90% and PSG23 92% down-regulation in KO mouse cervical tissue compared to WT mouse tissue. Black bars represent the control cells (KLF10ct) or the WT mouse (KLF10wt), while grey bars represent the KLF10 knock down cells (KLF10kd) and knock out mouse (KLF10ko) in each panel respectively. Panel (C1–C4) shows the *in silico* results for the analysis of promoter regions from IL6, IL25, PSG2 and PSG5 genes respectively, MatInspector module from Genomatix Software was used. Pink rectangles represent the putative region were KLF10 responsive sequence could be located. Panel (D) show the KLF10-immunoprecipitated DNA. The first two lanes present PSG promoter region associated to KLF10. Whereas lanes three and four present GAPDH promoter in RNA Pol II immunoprecipitated DNA as positive control, and PSG5 promoter in mouse-IgG immunoprecipitated DNA as negative control. All ChIP lanes present Input DNA control. Agarose gel was cropped; all reactions were electrophoresed in the same gel.

Given our findings of DNA copy number gains and the increased expression of the PSG genes and IL25, we next assessed the proximal promoter regions of the PSG2, PSG5 and IL25 genes for KLF10 binding sites. Interestingly, the proximal core promoter regions of the PSG2, PSG5 and IL25 genes contained at least two putative KLF binding sequences (Fig. 3). To explore this further, we knocked down KLF10 in the SiHa cervical cancer cell line and perform microarray analyses.

The SiHa cell line was chosen as an experimental model for KLF10 knock down because this cell line was established from a cervical squamous cell carcinoma that contains HPV16 sequences, most of CC cases have similar parameters. Once the validation of at least 70% down regulation of KLF10 protein expression was achieved (Fig. 3), we carried out microarray analysis to identify altered genes whose expression levels were altered due to suppression of KLF10 expression.

Genes previously described as transcriptionally regulated by KLF10 such as fibroblast growth factor receptor (FGFR), epidermal growth factor receptor (EGFR), TGFβ receptor II, and runt-related transcription factor (RUNX) among others showed down-regulation in the KLF10 knocked down cells. Supplementary Tables 1 and 2 shows the list of genes up- and down-regulated.

Pathway analysis of the microarray data using genes whose expression was significantly decreased by at least 1.5 fold indicated that KLF10 participates in several processes including the melanoma pathway and metabolism of xenobiotics by cytochrome P450 among others.

DAVID and WebGESTALT based gene pathway analysis showed that the most altered events were associated with cytokines. The down-regulated cytokines detected in the microarray analysis included IL6, IL17A, IL25 and the chemokines CXCL1, −11–13, CCL3, −11, −13, −16 among others (Fig. 3, Table 2). We also observed suppression of Arachidonate Lipoxygenase (ALOX) ALOXE3 and ALOX15, which are bioactive lipid metabolizing enzymes known to be associated with immune modulation.

**Table 2:** KEGG-based pathway analysis results from down-regulated genes in KLF10-siRNA treated SiHa cell line obtained from DAVID.

Category	Term	Count	Genes
KEGG_PATHWAY	hsa05218:Melanoma	13	EGFR, BRAF, FGF16, MITF, IGF1, CDH1, FGF12, RB1, PDGFC, FGF1, FGF2, PIK3R1, FGF4
KEGG_PATHWAY	hsa04810:Regulation of actin cytoskeleton	29	SSH1, APC2, WASF1, FGF16, IQGAP3, FGF12, ITGB3, DOCK1, TIAM1, ARPC2, ITGAV, ITGB6, PDGFC, FGF1, FGF2, PIK3R1, FGF4, APC, EGFR, BRAF, IGF2, PPP1CB, CHRM5, CFL2, CFL1, ITGA7, CYFIP1, CRK, MYH10
KEGG_PATHWAY	hsa00983:Drug metabolism	9	CYP3A5, NAT1, NAT2, CYP2A7, UGT2A3, HPRT1, UGT2B28, IMPDH1, UGT2B7
KEGG_PATHWAY	hsa04060:Cytokine-cytokine receptor interaction	32	CXCL1, IFNA21, CNTFR, CXCL11, TNFRSF1A, IL17A, IL1RAP, TPO, CSF2RB, PDGFC, IFNK, IFNA8, PRL, RTEL1, IFNGR1, EGFR, IL6, IL2RA, IL29, TGFBR2, IL25, LIFR, TNFRSF17, EDA2R, CCL4L1, CCL16, CCL11, IFNAR2, CCL13, CXCL13, NGFR, IFNA17
KEGG_PATHWAY	hsa00980:Metabolism of xenobiotics by cytochrome P450	10	CYP3A5, GSTM3, GSTM4, CYP2F1, ADH1B, GSTO1, UGT2A3, UGT2B28, UGT2B7, ALDH3A1
KEGG_PATHWAY	hsa04910:Insulin signaling pathway	18	BRAF, PHKB, PRKCI, PRKAB1, PDE3B, IGF2, RPS6, PPP1CB, IRS1, GCK, SLC2A4, PRKAR1B, PKLR, GYS1, SHC1, CRK, CALM2, PIK3R1

As expected, down-regulation of PSG2 and −5 were observed following suppression of KLF10 (Fig. 3). The genes down-regulated were not the only ones affected in relation to immune system response, the up-regulation of several genes involved in immune-related functions were also observed, that include IL7, IL17C, and IL32. Chemokine CCL14 and a number of cytokines and chemokines receptors were also up-regulated.

### Down-regulation of PSG and Interleukin mRNA in KLF10 KD cells

Altered expression of PSG2, PSG5 and IL25 following KLF10 knock down were selected for further validation because of their correlation with DNA gain in copy number and microarray expression patterns in CC, and their previous association with immune system response. Since IL6 is known to be up-regulated in CC^5^, we used this as a control to validate the microarray results. Of the transcripts analyzed, PSG2 was the most differentially expressed gene, both in the microarray data (−2.87) as well as in the RT-qPCR analysis with a down regulation of 89% in the KLF10 siRNA treated cells compared to control cells, IL25 was down-regulated by 52%, PSG5 by 43% and IL6 by 38% following knockdown of KLF10 (Fig. 3).

### Microarray results of cervical tissues of KLF10 knock out (KO) mice

Genes previously known as direct transcriptional targets of KLF10 including FGFR and RUNX exhibited down regulation in KO mouse cervical tissue, results that were similar to our KLF10 knocked down CC cell line (Supplementary Tables 3 and 4).

DAVID and WebGESTALT KEGG-based analyses of the down-regulated genes revealed that KLF10 participates in processes such as neuroactive ligand-receptor interaction, cancer related pathways as well as metabolism of xenobiotics by cytochrome P450, JAK-STAT signaling, cell adhesion molecules (CAM’s), cytokine-cytokine receptor interaction and the chemokine signaling pathways (Table 3).

**Table 3:** KEGG-based pathway analysis results from down-regulated genes in KLF10^−/−^ (KO) mouse cervical tissue obtained from DAVID.

Category	Term	Count	Genes
KEGG_PATHWAY	mmu03030:DNA replication	8	PRIM1, RFC3, RFC4, LIG1, POLE, POLA1, RNASEH1, RNASEH2B
KEGG_PATHWAY	mmu04630:Jak-STAT signaling pathway	18	GRB2, CSF2RB2, CTF1, PIK3CD, SOCS4, SOCS5, IL7R, CISH, IL11, IL6RA, LIF, SPRY1, ILTIFB, PIAS3, SOS1, SPRED2, PIK3CA, PIAS1
KEGG_PATHWAY	mmu04080:Neuroactive ligand-receptor interaction	26	F2RL2, AVPR2, C3AR1, THRB, TRHR, BDKRB1, FPR3, NR3C1, EDNRA, HCRTR1, HRH3, ADRA2A, TAAR1, CALCRL, HTR1D, GABRG2, GABRG3, CCKBR, PTH2R, TRHR2, NPBWR1, GABRR1, CHRM2, P2RY14, MTNR1B, MTNR1A
KEGG_PATHWAY	mmu04514:Cell adhesion molecules (CAMs)	17	CLDN16, SELP, PTPRC, ICOSL, H2-D1, CDH1, NCAM1, SIGLEC1, ITGB2L, CD80, ITGAV, PVRL2, H2-AA, CNTN1, VCAN, 4930468A15RIK, CD28
KEGG_PATHWAY	mmu05211:Renal cell carcinoma	10	CDC42, CUL2, GRB2, SOS1, PIK3CD, ARNT2, GAB1, TCEB2, PIK3CA, PAK1
KEGG_PATHWAY	mmu04722:Neurotrophin signaling pathway	15	GRB2, PIK3CD, NTRK3, MAGED1, CDC42, SOS1, MAP3K1, NTRK2, GAB1, PIK3CA, SORT1, NGFRAP1, SH2B2, MAPK7, CAMK2A
KEGG_PATHWAY	mmu04512:ECM-receptor interaction	10	CD47, LAMA3, CD36, CD44, NPNT, ITGAV, COL3A1, AGRN, ITGB3, COL11A2
KEGG_PATHWAY	mmu04540:Gap junction	10	GJD2, ADCY7, GRB2, SOS1, TUBA4A, GUCY1B2, MAPK7, PRKACB, PRKG1, TUBA1B
KEGG_PATHWAY	mmu04520:Adherens junction	9	PTPRJ, FGFR1, CDC42, IGF1R, PVRL2, CTNND1, CDH1, WAS, SNAI1
KEGG_PATHWAY	mmu00980:Metabolism of xenobiotics by cytochrome P450	8	GSTM3, CYP3A16, CYP1A1, CYP2B19, GSTZ1, CYP2B10, CYP2C39, CYP3A44
KEGG_PATHWAY	mmu04060:Cytokine-cytokine receptor interaction	19	CSF2RB2, TNFRSF25, CTF1, IL7R, CCL28, IL11, TNFSF8, IL6RA, LIF, CCR8, TNFRSF11B, ILTIFB, INHBC, IL1RAP, TNFRSF18, XCL1, BMP7, XCR1, EDA
KEGG_PATHWAY	mmu04062:Chemokine signaling pathway	14	ADCY7, GRB2, PIK3CD, CCL28, WAS, CCR8, CDC42, SOS1, IKBKG, PIK3CA, PAK1, PRKACB, XCL1, XCR1

Interestingly, immune response genes such as PSG17, −21, −23, IL11, interleukin 1 receptor-like 1 (IL1RL1), IL1RAP, chemokines CCR8, CCL28 and XCL1 (Fig. 3) were also down-regulated in KLF10 KO cervical tissues. Additionally, ALOX15, which was recently described to participate in the immune response in cancer, was also down regulated in KLF10 mice. Up-regulation of IL21, IL12B and cytokine and chemokine receptors as well as IL3RA, IL9R and CCR4 were observed. These results are in concordance with the results obtained in our cell line studies.

### Validation of PSG mRNA results in KLF10 KO mice

The PSG family members were chosen for RT-qPCR validation, as they are relatively novel molecules involved in cancer and pregnancy immune system regulation. As shown in Fig. 3, we observed that PSG17 was down-regulated 18%, PSG21 90% and PSG23 92% in KLF10 KO mouse cervical tissues compared to KLF10 WT controls (Fig. 3).

### KLF10 associates with PSG gene promoters

Given our results indicating decreased expression of PSG2 and −5 in SiHa cancer cells and cervical tissues following loss of KLF10 expression, and in light of the *in silico* analyses indicating putative KLF10 binding sites located in the promoter regions of these genes, we sought to determine if KLF10 indeed binds to these sites. Chromatin immunoprecipitations assays (ChIP) confirmed KLF10 binding to the PSG2 and PSG5 promoter regions. These results are consistent with our gene expression studies and provide further evidence that PSG2 and PSG5 are direct KLF10 target genes in the cervix (Fig. 3).

## Discussion

Genomic instability is manifested by gains and losses of chromosomal regions in cancer. Importantly, the recurrence of specific aberrations (both amplifications and deletions) at particular sites in the genome indicates that such sites are likely to harbor genes whose alteration favors neoplastic progression^2^. Along with DNA alterations, transcriptomic analysis and particular mRNA expression profiles could indicate which molecular pathways are altered and contribute to disease development and progression. In the present study, we have identified novel molecular markers that are likely to play important roles in CC and its immune regulation. Although many genes were identified, KLF10 was a transcription factor that exhibited copy number amplification and which exhibited high expression levels in cervical cancer biopsies^10^. This gene is known to play an important role in immune response and TGFβ signaling pathway^10^. Copy number amplification of this gene has also been reported in lung cancer^15^.

Low-Grade squamous intraepithelial lesions exhibit slightly altered patterns of differentiation and are frequently cleared by the immune system in less than a year, however, some of these lesions can persist and progress to CC^3^.

Immune system response, both pro- and anti- inflammatory processes, has recently been recognized as part of the “hallmarks of cancer” and plays a pivotal role in the development and progression of transformed cells^2^. It has been reported that there is an imbalance in the Th1/Th2/Th17 immune responses that are associated with differential expression of anti- and pro- inflammatory cytokines in CC^16,17^.

By a broad definition, inflammation involves tissue-remodeling events that are associated with alterations in epithelial, vascular and immune cell functions^18^. Specific molecular pathways involving a host of transcription factors, cytokines, chemokines, growth factors and lipid mediators orchestrate these events. KLF family members have been implicated in the function and differentiation of immune cells^19^.

The up-regulation of IL6 in CC has been reported^20^ to promote the expression of vascular endothelial growth factor (VEGF) via the STAT3 pathway, and therefore possibly mediates angiogenesis^21^ and autocrine growth factor signaling^22^. In light of immune cell skewing, IL6 has been shown to promote Th2 differentiation by induction of IL12, to inhibit Th1 differentiation by suppression of IFNγ, and participates in B cell and macrophages differentiation^23^ and probably recruitment of Th17 cells^24^.

IL25 (IL17E) belongs to the IL17 family and has been implicated in the expression of NF-κβ, activation of MAPK, as well as promotion of eosinophilic infiltration, mucus production and epithelial cell hyperplasia^25^. It is also involved in Th2 and Th9 differentiation^26^. To our knowledge this is the first report indicating IL25 up-regulation in CC, although its role in this disease is not yet fully understood. IL25 was not the only member of the IL17 family affected by down-regulation of KLF10 as IL17A also exhibited decreased expression at the mRNA level in the present study. This gene has been shown to be over expressed in CC and plays a critical role in migration by induction of matrix metalloproteinase 2 and −9 trough p38/NF-κβ pathway^27^ and regulates angiogenesis by induction of VEGF^28^.

On the other hand, Pregnancy-Specific Beta-1 Glycoproteins comprise a family of 10 genes (PSG1–9, and −11) and one pseudogene (PSG10) clustered in the 19q13.31 cytogenetic region. They are members of the carcinoembryonic antigen family^29^. Our CNV results show a gain in copy number of the 19q13.31 cytogenetic region, were the PSG gene cluster is encoded. Here we report the up-regulation of two members, PSG2 and PSG5, and to our knowledge this is the first report showing up regulation of PSG mRNA molecules in CC. Members of the PSG family have been shown to be up-regulated in breast and gastric cancers^30,31^. The members of this family have also been implicated in angiogenesis as well as tubulogenesis by two possible mechanisms, one by the regulation of TGFβ and the other by interaction with glycosaminoglycans^29^. The PSG family members are also involved in the differentiation of Th1, Th2, Th17 and Treg cells by increasing the expression of cytokines such as IL6, −10, −17 and TGFβ^32,33^.

Through the use of *in silico* analyses, we have demonstrated that IL6, IL25 and PSG genes are potential targets for KLF10 since they possess KLF binding sites in their proximal promoters. This possibility was confirmed by ChIP assay demonstrating recruitment of KLF10 to these putative binding sites. There is evidence that KLF10 acts as a transcriptional regulator of cytokines and chemokines including IL12p40, TGFβ^11^ and possibly IL6^13^ CCR7 as well as the receptor CXCR4^34^. There is also clear evidence that other members of the KLF family can regulate the transcription of PSG genes^29^. It has been shown that CC derived cells exhibit deregulation of IL6, IL17A and IL17E which subsequently influencing leukocytosis^35^. All of these CC derived cell lines also express KLF10, as evidenced from our findings. Similarly, there are reports demonstrating that KLF10^−/−^ mice have higher expression of cytokines that influence the Th1 and Th2 differentiation profiles^9^. Our results support the possibility that KLF10 plays an important role in regulating essential genes such as PSG2 and −5 in CC and also plays a critical role in immune modulation within the cervix. Given that KLF10-specific small molecule inhibitors have been developed and are known to elicit potent effects in T cells^36^, such drugs could represent a novel treatment for CC.

## Conclusions

Here, we demonstrate that KLF10 plays important roles in the transcriptional regulation of genes involved in the immune system response including IL6, IL25 and members of the PSG family. Regulation of these genes is important with regard to the immunoediting process in cervical tissue and likely contributes to CC development and progression. The gain in DNA copy number for the 8q22.3 and 19q13.31 cytogenetic regions represent potential DNA molecular markers in CC. The increased expression of IL25, PSG2 and PSG5 could also serve as molecular markers and potential drug targets for immune-based therapies in CC, and may also play important roles in the balance of the immune system response in CC.

## Materials and Methods

### CNV *in silico* analysis, data mining and analysis

For copy number variation analysis, a total of 115 cancer libraries and 32 control libraries corresponding to GSE10092 and GSE52904 were downloaded and quality control checked prior to analysis. Partek v6.6 (Partek Incorporated, Saint Louis, MO, USA) were used with stringent parameters set as follows: each segment must contain a minimum of 10 consecutive filtered probe sets, a p-value threshold of 0.001 when compared to the neighboring adjacent regions and a signal-to-noise threshold of 0.5. The cut-off value for the gain was set at above 2.3, while loss was set at below 1.7. CNV was called for the gains or losses that occurred in at least 10% of the total samples. The 22 somatic chromosomes and the X chromosome were analyzed. Hierarchical clustering was performed using the following parameters: sample dissimilarity: Euclidian and cluster method: average linking.

### *In silico* analysis of the cervical cancer transcriptome

A total of 8 normal (N) and 57 cervical cancer (CC) experiments were downloaded and analyzed. The data used for these analyses were downloaded from European Bioinformatics Institute (EMBL-EBL) and GEO. These data correspond to GSE7307, GSE5787, GSE3526 and 1 Gene Expression Atlas (GSE2109) corresponding to Affymetrix Human Gene Chip U133 platform. Quality control checks were performed on all transcriptomic libraries downloaded, prior to inclusion in the analysis.

Data sets were analyzed by means of CEL files with the Expression Console, Partek Genomics Suite 6.6 v software (Partek Incorporated, Saint Louis, MO, USA) and Transcriptome Analysis Console (Affymetrix, Santa Clara, CA, USA). Pearson and Spearman correlation was performed and probe sets were summarized by means of Median Polish and normalized by quantiles with no probe sets excluded from analysis. Background noise correction was achieved by means of Robust Multi-chip Average (RMA) and data were log2- transformed. Data grouping and categorization was achieved by principal component analysis (PCA). Differentially expressed genes were determined by means of ANOVA. Genes were considered altered with +1.5 or −1.5 fold change, *p* ≤ *0.05* and FDR >0.05 parameters.

### Cervical tissue collection

For confirmation of the *in silico* findings, we collected three histologically normal tissues and twenty carcinomas. Patients were recruited with signed informed consent and ethical approval from the Comisión Nacional de Ética e Investigación Científica del Instituto Mexicano del Seguro Social in accordance with the Helsinki declaration. These same cervical tissues have been previously described^10^.

### KLF10 knock out and wild type mice

Eight weeks old female KLF10−/− knockout (KO) and KLF10+/+ wild-type (WT) mice were used for isolation of the cervical tissues that were used in the microarray experiments. The KLF10 KO mice used in this study were generated as described previously^37^. This study was carried out in strict accordance with the recommendations in the Guide for the Care and Use of Laboratory Animals of the National Institutes of Health. The protocol was approved by the Mayo Clinic Institutional Animal Care and Use Committee.

### Cell culture and siRNA treatment

All reagents used for the cell culture and siRNA transfection were purchased from Life Technologies (Foster City, USA) unless otherwise stated.

The SiHa cell line was cultured in Dulbecco’s Modified Eagle Medium (DMEM) supplemented with 10% fetal bovine serum (FBS) and 1% penicillin/streptomycin at 37 °C in 5% CO_2_ in 12 well plates. Transient transfection was performed using KLF10-specific Silencer Select siRNA: s14129 and s14128, and Lipofectamine RNAiMAX reagent was used for transfection. Briefly, approximately 90’000 SiHa cells per well were seeded and cultured overnight. Seventy nM siRNA was diluted in Opti-MEM media generating solution 1, Lipofectamine RNAiMAX was diluted in Opti-MEM media generating solution 2. Solution 1 and 2 were mixed at equal volumes and incubated at room temperature for 20 min, following the manufacturer’s protocol, and the mixture was added to SiHa cells in a total volume of 500 μl. As a negative control, a scrambled siRNA (AM4611) was transfected with the same conditions as the KLF10 siRNA mentioned above. A GFP expression construct was used as a positive control. For comparison purposes, SiHa cell cultured in the absence of any treatment were also examined. Six hours following transfection, 500 μl of DMEM supplemented media was added to the cell culture. All treatments were performed in triplicate a minimum of three independent times. Forty-eight hours post transfection; RNA was isolated, pooled and used for microarray experiments.

### Hybridization and analysis of microarray data

#### Printing of arrays

Human 70-mer oligo library from OPERON Oligo Sets (http://omad.operon.com/) was resuspended to 40 μM in Micro Spotting solution (Telechem International Inc.). SuperAmine coated slides, 25 × 75 mm (TeleChem International INC) were printed in single copy, and fixed at 80 °C for 4 hours. For pre-hybridization, the slides were re-hydrated with water vapor at 60 °C, and fixed with two cycles of UV light (1200 J). After boiling for two minutes at 92 °C, slides were washed with 95% ethanol for one minute and prehybridzed in 5 × SSC, 0.1% SDS and 1% BSA for one hour at 42 °C. The slides were washed and dried prior to hybridization.

#### Probe preparation and hybridization to arrays

RNA integrity was analyzed both by agarose gel electrophoresis and by a Bioanalyzer 2100 and only samples with RNA Integrity Numbers (RIN) greater than 8 were used in these assays. 10 μg of total RNA were used for cDNA synthesis incorporating dUTP-Alexa555 or dUTP-Alexa647 employing the CyScribe First-Strand cDNA labelling kit (Amersham). Incorporation of fluorophore was analyzed by absorbance at 555 nm for Cy3 and 655 nm for Cy5. Equal quantities of labeled cDNA were hybridized using hybridization solution HybIT2 (TeleChem International INC). The arrays were incubated for 14 h at 42 °C, and then washed tree times with 1 × SCC, 0.05% SDS at room temperature. Cy3 labeling was used for control SiHa control cells and WT mouse tissue while Cy5 labeling was used for KLF10 siRNA-treated cells and KO mouse tissue.

#### Data acquisition and analysis of array images

Acquisition and quantification of array images was performed in ScanArray 4000 with its accompanying software ScanArray 4000 from Packard BioChips. All images were captured using 65% PMT gain, 70 to 75% laser power and 10 µm resolution at 50% scan rate. For each spot the Cy3 and Cy5 density mean value and the Cy3 and Cy5 background mean value were calculated with software ArrayPro Analyzer from Media Cibernetics.

#### Data analysis

Microarray data analysis was performed with free software genArise, developed in the Computing Unit of Cellular Physiology Institute of UNAM (http://www.ifc.unam.mx/genarise/). GenArise carry out a number of transformations: background correction, Lowess normalization, intensity filter, replicates analysis and selecting differentially expressed genes. The goal of genArise is to identify which of the genes show good evidence of being differentially expressed. The software identifies differential expressed genes by calculating an intensity-dependent z-score. Using a sliding window algorithm to calculate the mean and standard deviation within a window surrounding each data point, and define a z-score where z measures the number of standard deviations a data point is from the mean.

1 $${{\rm{z}}}_{{\rm{i}}}=({{\rm{R}}}_{{\rm{i}}}-{\rm{mean}}({\rm{R}}))/{\rm{sd}}({\rm{R}})$$

where z_i_ is the z-score for each element, R_i_ is the log-ratio for each element, and sd(R) is the standard deviation of the log-ratio. With this criterion, the elements with a z-score >1.5 standard deviations would be the significantly differentially expressed genes.

Furthermore, WebGESTALT (http://bioinfo.vanderbilt.edu/webgestalt/) and DAVID (http://david.abcc.ncifcrf.gov) were used for identifying and understanding the biological pathway and processes that are significantly altered based on the gene expression profiles. These analyses were conducted using KLF10 target genes that were significantly down regulated in KLF10 siRNA treated cells or KLF10 KO tissues.

### RNA extraction, reverse transcription reaction and RT-qPCR

RNA was extracted from SiHa siRNA-treated and control cells, mouse KO and WT cervical tissue and the cervical cancer biopsies from patients using the RNAeasy Mini Kit (Qiagen Inc, CA, USA) according to manufacturer’s instructions. RNA was quantified using a Nanodrop-ND-1000 (Thermo Scientific, DE, USA) and RNA integrity was evaluated on 1.5% agarose gel and by a Bioanalyzer 2100. After purification, 1 μg of total RNA was reverse transcribed in a 20 μl final volume reaction with the High capacity RNA-to-cDNA Kit (Applied Biosystems, Foster City, USA), 11 μl of Mix were added, and the reaction mixture was incubated at 37 °C for 60 min., and 95 °C for 5 min., and held at 4 °C indefinitely according to manufacturer protocols. For RT-qPCR of human PSG2, PSG5, IL6, Il25 and mouse PSG17, PSG21 and PSG23 all reagents were purchased from Applied Biosystems (Foster City, USA), and conditions were as follows: 11 μl of Taqman Gene Expression Master Mix, 1 μl of each Taqman probe, 500 ng of cDNA in a 20 μl final volume, according to manufacturers recommendation. RLPO and GAPDH for human and mouse respectively, were used as internal controls and all reactions were conducted in triplicate in the Step one thermal cycler (Applied Biosystems). 2−ΔΔCt was used to calculate relative expression levels. Probe Id’s are listed in Supplementary Material and Methods Table 1.

### Chromatin immunoprecipitation for KLF10 binding sites in PSG promoters

Chromatin immunoprecipitations (ChIP) were performed using the EZ-ChIP kit (#17–371 Millipore, Darmstadt, Germany) according to the manufacturers instructions. Briefly, 70% confluent SiHa cells were fixed with 1% formaldehyde for 10 min at room temperature and quenched with 10× glycine for 5 min. After quenching, cells were scraped in 1 × PBS containing Protease Inhibitor Cocktail II, pelleted and the supernatant was discarded. Cell pellets were re-suspended in SDS Lysis Buffer containing Protease Inhibitor Cocktail II. Cells were sonicated on ice using Sonics Vibracell VCX130 (Sonics, CT, USA). After sonication cell debris was eliminated by centrifugation at 12 000 g for 10 min at 4 °C. After this step, 900 μl of Dilution Buffer containing Protease Inhibitor Cocktail II was added to each 100 μl of sheared crosslinked chromatin. Pre-clearing process was carried out by incubating with 60 μl of the Proteing G Agarose beads for 1 h at 4 °C. The Protein G Agarose beads were collected by centrifugation at 4 000 g for 1 min at 4 °C. And 10 μl was collected as the Input loading control. Immunoprecipitation was carried out using antibodies against RNA Pol II (positive control), mouse IgG (negative control), both provided by the EZ-ChIP kit manufacturer. KLF10 specific antibodies (GTX108661; Genetex, CA, USA) and 992 (kindly donated by Drs. Subramaniam and Hawse, Mayo Clinic, USA) were also used. Immunoprecipitations were carried out via overnight incubation at 4 °C with primary antibodies. Following incubation, 60 μl of Protein G Agarose beads was added and incubated for 1 hr at 4 °C, and Antibody-Antigen-DNA was collected by centrifugation at 4 000 g for 1 min at 4 °C. Beads were washed by suspending them and incubating them for 5 min at 4 °C in Low Salt Immune Complex Wash Buffer one time, High Salt Immune Complex Wash Buffer one time, LiCl Immune Complex Wash Buffer one time and TE Buffer two times. Complexes were eluted two times in 100 μl of elution buffer (10 μl 20% SDS, 20 μl de 1 M NaHCO_3_ and 170 μl of water) and complex were suspended and incubated for 15 min at room temperature followed by centrifugation at 4 000 g for 1 min at 4 °C. Supernatant was collected and transferred to a new tube, resulting in a final elution volume of 200 μl. To reverse DNA-Protein crosslinks, 5 M of NaCl was added and samples were incubated overnight at 65 °C. Followed by addition of 1 μl of RNAse A and incubation at 37 °C for 30 min, to deplete al RNA species. For DNA extraction, 4 μl of 0.5 M EDTA, 8 μl of 1 M Tris-HCl and 1 μl of Proteinase K were added, and incubated for 4 hrs at 45 °C. DNA purification was performed using columns and reagents provided by the kit. Binding Buffer A, 1 ml for every 200 μl of elution buffer, was added, and 600 μl transferred to the column, centrifuged 12 000 g for 30 sec, this step repeated two times, until the 1.2 ml were passed through the column. Column was washed with 500 μl of Washing Buffer B and centrifuged 12 000 g for 30 sec. Centrifugation at 12 000 g for 30 sec was repeated to eliminate the excess of buffers in the column. DNA was eluted in 50 μl of Elution Buffer C by centrifugation at 12 000 g for 30 sec.

To evaluate candidate gene-promoters PCR was conducted as follows; 12.5 μl of GoTaq® Green Master Mix (Promega, WI, USA), 5 μl of ChIP-DNA template, and 20 pmol of each primer, in a 25 μl total volume reaction, with the following program: 30 seconds at 95 °C, 30 seconds at 59 °C and 30 seconds at 72 °C for 32 cycles.

The GAPDH promoter was analyzed in the ChIP assays as a positive control (RNA Pol II Ab), Pregnancy-Specific Beta-1 Glycoproteins 2 and 5 gene promoters were evaluated in KLF10 ChIP and IgG ChIP (Negative control) samples. Sequences of corresponding primers are listed in Supplementary Material and Methods Table 1.

### Statistical analysis

Clinical and pathological relation analysis was performed by means of the Student’s t-test for the gene expression studies and for clinical and pathological parameters that were normally distributed in the data sets. For non-normally distributed parameters, the Mann-Whitney U test was utilized. All p-values represent two-tailed tests and were considered significant at 0.05. Statistical analysis was performed using SPSS v15 statistical software.

## Electronic supplementary material

Suplementary Information 

supplementary lists 3 and 4

supplementary materials and methods
